# Writing for the Journal: A Guide for Community-Based Organizations

**DOI:** 10.13023/jah.0601.01

**Published:** 2024-09-01

**Authors:** Randy Wykoff, Rachel E. Dixon

**Affiliations:** East Tennessee State University; Journal of Appalachian Health

**Keywords:** Appalachia, academic writing, community-based organizations, journal submissions

## Abstract

The *Journal of Appalachian Health* welcomes submissions from a variety of stakeholders interested in and contributing to improvement of health across the Appalachian Region. This editorial provides basic guidelines for those working in community settings who may with to make *JAH* (or any other journal) their publication home.

## INTRODUCTION

There are many outstanding guidelines on how to write an article for publication in a medical, scientific, or public health journal. Most of these are developed for those working in academia—the group that submits the vast majority of articles to these journals.

Because so much important health-related work in Appalachia is done *outside* of academic centers, the *Journal of Appalachian Health (JAH)* actively encourages submissions from those working for community-based organizations, state and local governments, faith-based institutions, and other non-profit groups. Unfortunately, much outstanding work done by these groups goes unstudied and, even more commonly, unreported.

This brief editorial provides basic guidelines for those working in community settings who may wish to make *JAH* their publication home:

1. **Appreciate the wider value of your work**. Community-based organizations often do not consider publishing their work. Yet such organizations have important lessons and ideas to share! If you are implementing a new project, making significant changes to an old project, or even trying a proven approach in a new area, consider publishing it. If other people could learn from your experience, it is worth writing it up. Publishing not only informs your colleagues about what might (or might not) work in their area, it also enhances awareness of your organization and its work—both publicly and among potential funders.
2. **Think about evaluation from the start of any project**. Community-based organizations often fail to build evaluation into their projects early enough. This can be because monitoring and measurement are overlooked, tacked on at the end of projects, or felt to be too onerous. Start by asking yourself a simple question: *How will I know that my project has been successful?* Then, consider how you might go about measuring those indicators of success. Before you begin, consider reaching out to your local academic institution or to your state department of health; someone there may be able to guide you in establishing a monitoring and evaluation plan or even with the nitty gritty of measurement and statistics. Pay special attention to your inclusion criteria (i.e., how people are selected for your project); especially any aspect of your work that is so unique that it cannot be replicated elsewhere; and, of course, be sure that you have enough participants in your project to draw reasonable conclusions.
3. **Follow the correct research protocol**. Remember that research projects that involve people, that use identifiable information, or that are intended for publication require approval by an Institutional Review Board (IRB). The Federal Government maintains a list of available IRBs,^1^ but you can also reach out to a nearby academic institution.
4. **Structure your writing to align with the journal’s style**. Before you start writing, look at the guidelines and requirements of the journal. Do they want a structured abstract? (*JAH* requires this for research articles, but not for other pieces.) What is the word count limit? (This often varies by submission type.) Is there a cap on the number of figures, tables, or references? You will save yourself—and the journal editors!—a lot of time if you investigate and follow these requirements beforehand. Does this journal publish articles in other formats other than the traditional research report? (For example, JAH offers *Notes from the Field*, an opportunity to publish brief reports about on-going community-based work or promising early results.) For journals that publish free, open-access content online, such as *JAH*, it is a great idea to read a few published articles to get a better sense of content, structure, and style.
5. **Write the abstract first**. This will help guide you to tell your story succinctly and clearly. In general, “less is more.” Keep the entire article brief and clear so that it appeals to a large readership.
6. **Write well!** True, this is easier said than done; but there are strategies to help you improve if, as for many of us, your writing skills remain a work in progress. First, aim to keep language concise and free from obvious errors by editing after you put pen to paper. Several tried-and-true handbooks on writing can guide you to brevity, better grammar, and proper spelling, should you need them.^2^,^3^,^4^ It also helps to have a trusted colleague read your article before you submit. Finally, you can revisit other published articles to see what appeals to readers—and what does not.
7. **Following the initial submission, have patience**. It takes weeks, and sometimes months, to get peer reviews in hand—and that is only the first step towards making revisions. The editors will reach out when comments are ready.
8. Finally, **don’t take rejections or reviewers’ comments personally**. If you do get rejected, take the reviewers’ comments into account and, if appropriate, revise your manuscript and submit it to another journal.

If you have any questions about a potential submission, please reach out to us. We would welcome the chance to talk with you about your work.

## References

[1] Office for Human Research Protections [OHRP] Database for Registered IORGs & IRBs, Approved FWAs, and Documents Received in Last 60 Days U.S. Department of Health and Human Services 2024 Available at: https://ohrp.cit.nih.gov/search/irbsearch.aspx?styp=bsc Accessed Aug. 6, 2024

[2] Moran J First you write a sentence: The elements of reading, writing and life New York Penguin 2019 13:978-2-41-97851-1

[3] Cutts M The Oxford guide to plain English 5th ed Oxford UK Oxford University Press 2020 13:978-0-19-884461-7

[4] Zinsser W On writing well: The classic guide to writing nonfiction 30th anniversary ed, 7th ed New York Harper Collins 2006 13:978-0-06-089154-1

